# Altered Levels of Toll-Like Receptors in Circulating Extracellular Vesicles in Multiple Sclerosis

**DOI:** 10.3390/cells8091058

**Published:** 2019-09-10

**Authors:** Pavan Bhargava, Carlos Nogueras-Ortiz, Sahil Chawla, Rikke Bæk, Malene Møller Jørgensen, Dimitrios Kapogiannis

**Affiliations:** 1 Department of Neurology, Johns Hopkins University, Baltimore, MD 21287, USA; 2 Laboratory of Neurosciences, National Institutes of Aging, Baltimore, MD 21225 USA; 3 Department of Clinical Immunology, Aalborg University Hospital, 9000 Aalborg, Denmark

**Keywords:** multiple sclerosis, extracellular vesicles, TLR3, TLR4, innate immunity

## Abstract

Extracellular vesicles (EVs) are involved in inter-cellular communication and their cargo may provide prognostic/diagnostic biomarkers. To discover EV-associated biomarkers for Multiple Sclerosis (MS), we used an immune marker array to identify surface proteins on circulating EVs that differ between MS patients and controls (n = 3 each). We identified toll-like receptor-3 (TLR3) as a potential target for further validation. We utilized prospectively collected serum from relapsing-remitting MS patients (n = 18) and controls (n = 16) and confirmed lower concentration of TLR3 and higher concentration of mechanistically related TLR4 in MS EVs compared to controls. Future studies may further evaluate the utility of EV-associated TLRs as MS biomarkers and uncover their mechanistic significance.

## 1. Introduction

Multiple sclerosis is a chronic autoimmune disorder of the CNS with inflammatory and neurodegenerative components [1]. There is a need for both diagnostic and prognostic biomarkers to help improve the management of this heterogenous disorder. Extracellular vesicles (EVs) are membranous nanoparticles produced by all cells and contain cargo of nucleic acids (RNA, DNA), proteins and metabolites, whereas their surface is replete with immune mediators. Based on their cell of origin and cargo, EVs can have a variety of functions including cellular homeostasis and inter-cellular communication [2,3]. In neurological disorders such as Alzheimer’s disease (AD), Parkinson Disease (PD), and Neuro-HIV, the contents of EVs have been demonstrated to provide diagnostic, prognostic and therapeutic response biomarkers [4,5,6].

Alterations in EVs were described in MS almost two decades ago [7]. Previous studies in MS have largely examined the miRNA content of circulating EVs and have demonstrated significant alterations in miRNA profiles and relationships to disease course and treatment response [8,9,10]. Specific miRNA have also been implicated in potentially pathogenic effects on the immune system in MS [11]. MS EVs also appear to differ from controls in their lipid content [12,13]. Only one previous study evaluated the protein cargo of circulating EVs and noted alterations in myelin associated proteins [14]. While alterations in both the adaptive and innate immune system play critical roles in MS, the expression of immune markers has not been evaluated in MS EVs.

In this study, we first employed an extensively utilized EV Array targeting surface markers including immune mediators [15,16] and identified potential MS biomarker targets that were subsequently validated in a larger cohort of MS and control participants using targeted immunoassays. We demonstrated lower toll-like receptor 3 (TLR3) concentration and higher concentration of the mechanistically related TLR4 in circulating EVs of MS patients as compared to healthy controls.

## 2. Materials and Methods

### 2.1. Participants, Standard Protocol Approvals, and Patient Consents

We enrolled patients with Relapsing Remitting (RR) MS from the Johns Hopkins MS Center and age-, sex-, and race-matched controls. The protocol for this study was approved by the Johns Hopkins Institutional Review Board. All participants provided informed consent. Demographic and disease related information was collected from all participants. Participants underwent phlebotomy and blood was collected in red-top tubes and allowed to clot for 30 min, followed by centrifugation at 3000 RPM for 10 min to separate serum. Serum was then divided into 1.0 mL aliquots, which were stored at −80 °C until the time of EV isolation.

### 2.2. Isolation of Extracellular Vesicles

Serum samples were thawed on ice and 500 μL were immediately subjected to commercially available nanoparticle precipitation solution Exoquick^®^ (System Biosciences, Mountainview, CA, U.S.) to isolate total EVs. First, the total EVs were sedimented according to the manufacturer’s instructions, and then resuspended in 200 μL of ultra-pure distilled water supplemented with protease and phosphatase inhibitors. 10 μL of intact EVs suspension were used for the determination of particle concentration and diameter using nanoparticle tracking analysis (NTA) (Nanosight NS500, Malvern, Amesbury, UK) and (for 6 samples) 10 μL of intact EVs were used for the EV Array. For the NTA, the particle concentration in function of particle diameter was averaged from five 20-s videos captured using the following settings: scattering camera level, 15; slider shutter, 1206; slider gain, 366; detection threshold, 3; and sample dilution ranging from 1:100 to 1:200 providing a range of 50–250 particles/frame. The remaining volume was mixed with 300 μL of MPER lysis buffer (Thermo Scientific, Grand Island, NY, U.S.) supplemented with protease (cOmplete ULTRA Tablets, Roche, Millipore Sigma, St. Louis, MO, U.S.) and phosphatase (HALT Phosphatase Inhibitor Cocktail, Thermo Fisher Scientific, Cincinnati, OH, U.S.) inhibitors and was subjected to two freeze-thaw cycles to generate lysates for downstream ELISA.

### 2.3. EV Array

The EV Array was developed based on the technology of protein microarray to phenotype serum-derived EVs (optimized for small EVs/exosomes) for multiple antigens without any enrichment or purification prior to analysis [16]. Micro-sized spots of 40 different capturing antibodies (200 µg/mL) against known EV surface antigens were printed on standard epoxysilane-coated microarray slides (SCHOTT Nexterion, Germany) using a SpotBot^®^ Extreme Protein Edition Microarray Printer (ArrayIt Corporation, CA, U.S.). The EV Array was performed as described by Jørgensen et al., 2013 with modifications. In short, the microarray slides were initially blocked (50 mM ethanolamine, 100 mM Tris, 0.1% SDS, pH 9.0) prior to incubation with 10 µL serum sample diluted (1:10) in wash-buffer (PBS/0.05% Tween®20). The incubation was performed in Multi-Well Hybridization Cassettes (ArrayIt Corporation) at RT for two h, followed by overnight incubation at 4 °C. A cocktail of biotinylated detection antibodies (anti-human-CD9, -CD63, and -CD81, Ancell, MN, U.S.) diluted 1:1500 was used to detect retained EVs using Cy5-labelled streptavidin (Life Technologies, MA, USA) diluted 1:1500. Scanning and spot detection was performed as previously described (Bæk and Jørgensen, 2017). The data from the EV Array were compared as raw values and also normalized by the total number of particles present in 10 μL (measured by NTA). Afterwards, the data were log2 transformed; therefore, biomarker differences can be considered as “per EV”. The relation between positive and negative controls was determined to be >0.98, suggesting an acceptable level of background noise for all measured markers.

### 2.4. Measurement of Markers in EVs

EV Array results for proteins differentially expressed in MS patients and controls were validated by colorimetric ELISA using total EV lysates (TLR3: cat. no. ab131557, Abcam, Cambridge, MA, U.S.; TLR4: cat. no. LS-F3675, LSBio, Seattle, WA, U.S.). First, we determined the optimal sample dilution providing signals within the linear range of each standard curve using a subset of samples. Samples were assessed either undiluted or diluted in 1:1 and 1:4 ratios using the buffers provided. Based on results, we proceeded to evaluate TLR3 and TLR4 protein levels in the entire relapsing-remitting multiple sclerosis (RRMS) discovery cohort using samples diluted in a 1:1 ratio. Each sample was assessed in duplicate obtaining mean coefficients of variance of 4.6% for TLR3 and 3.0% for TLR4. Protein concentration was extrapolated from raw absorbances using the trendline equations of standard curves from which the limit of detection (LoD) and lowest limit of quantification (LLoQ) were determined. The LoD was defined as the mean signal of the diluent blank plus 2.5 times its standard deviation, whereas the LLoQ was determined by the standard solution with signal above the LoD, CV among duplicates lower than 20%, and recovery between 80% and <120%. Samples with protein concentration below the LLoQ but with signal above the LoD and CV <15% were assigned the plate LLoQ.

### 2.5. Statistical Analysis

We compared groups for demographic characteristics using either a *t*-test for continuous or a chi-square test for categorical variables. We compared concentrations of markers in the EV Array using a Welch’s *t*-test and performed correction for false discovery using a Benjamini-Hochberg procedure setting the false discovery rate at 0.2. For the TLR3 and TLR4 concentrations, we used a Mann-Whitney U-test to compare groups, given the sample size. We considered a *p*-value of <0.05 as significant. For the individual marker comparisons, we also excluded outlier values (defined as 1.5 times the interquartile range [IQR]) above the third quartile or below the first quartile—this resulted in removal of two observations in each group for TLR3 and two observations in the healthy control (HC) group and one in the MS group for TLR4.

## 3. Results

### 3.1. Study Cohort

We enrolled 18 RRMS patients and 16 controls in the study. The demographic and disease characteristics are summarized in Table 1. There were no significant group differences in age, sex, or race. The majority of RRMS patients were either untreated or on an injectable disease-modifying therapy (interferon-beta or glatiramer acetate), however, two participants were receiving natalizumab.

### 3.2. EV Array Identified Lower Levels of Multiple Surface Markers in MS Compared to Controls

In an initial experiment involving a small set of MS patients and controls (n = 3 each), we utilized the “EV Array” to detect the presence of various surface markers and immune mediators on intact serum EVs. Of the three MS patients chosen for the EV array, one was untreated, one was on glatiramer acetate, and the third was on natalizumab. We noted a reduction or trend for several markers, with significant differences listed in Table 2. We chose TLR3 as a target for further validation since it was one of the most differentially expressed markers and has been known to play important roles in innate immunity [17]. For mechanistic context, we decided to also assess the abundance of a TLR4, a receptor not included in the EV Array that is also associated with innate immunity and implicated in several inflammatory disorders, including MS [18,19,20], often with opposing activity to TLR3.

### 3.3. TLR3 and TLR4 Levels Are Altered in MS Patient EVs

**Figure 1:**
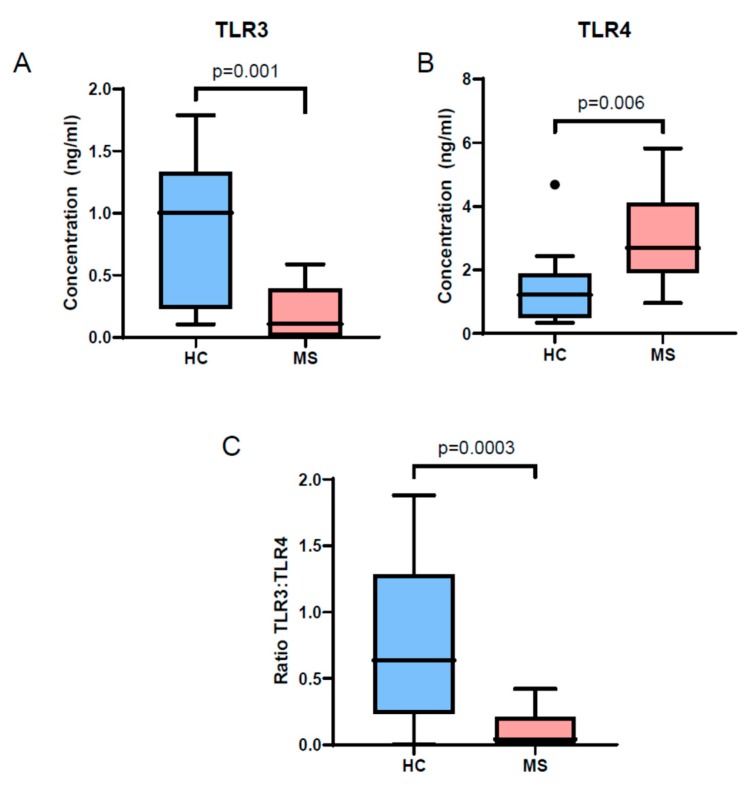
Toll-like receptor-3 (TLR3) is reduced and TLR4 is increased in EVs from MS patients compared to controls. We compared the levels of TLR3 and TLR4 in EVs isolated from MS patient serum. (**A**) We noted reduced concentration of TLR3 in EVs from the MS group (n = 18) compared to controls (n = 16). (**B**) We also noted increased concentration of TLR4 in EVs from the MS group compared to controls. (**C**) The ratio of TLR3 to TLR4 in EVs was also reduced in the MS group compared to controls. All comparisons were made using a Mann-Whitney U test.

Next, we isolated EVs from serum samples from the entire cohort and performed quantification of TLR3 in lyzed total EVs. Confirming the EV Array results, we noted reduced levels of TLR3 in MS patient EVs compared to controls (Figure 1A). We also measured TLR4 and noted higher TLR4 levels in MS patient EVs compared to controls (Figure 1B). We also calculated the ratio of TLR3:TLR4 concentration and noted that the ratio was significantly lower in MS patients compared to controls (Figure 1C).

Since, we had patients with varying treatment status, to exclude an effect of treatment on the results of TLR3/4 levels in EVs, we performed a sensitivity analyses by comparing levels of TLR3 in EVs between MS patients grouped based on treatment status (either on a disease modifying treatment or no DMT) and HCs. We noted that both MS sub-groups had significantly lower levels compared to healthy controls (no DMT-p = 0.031, on DMT-p = 0.023).

**Figure 2:**
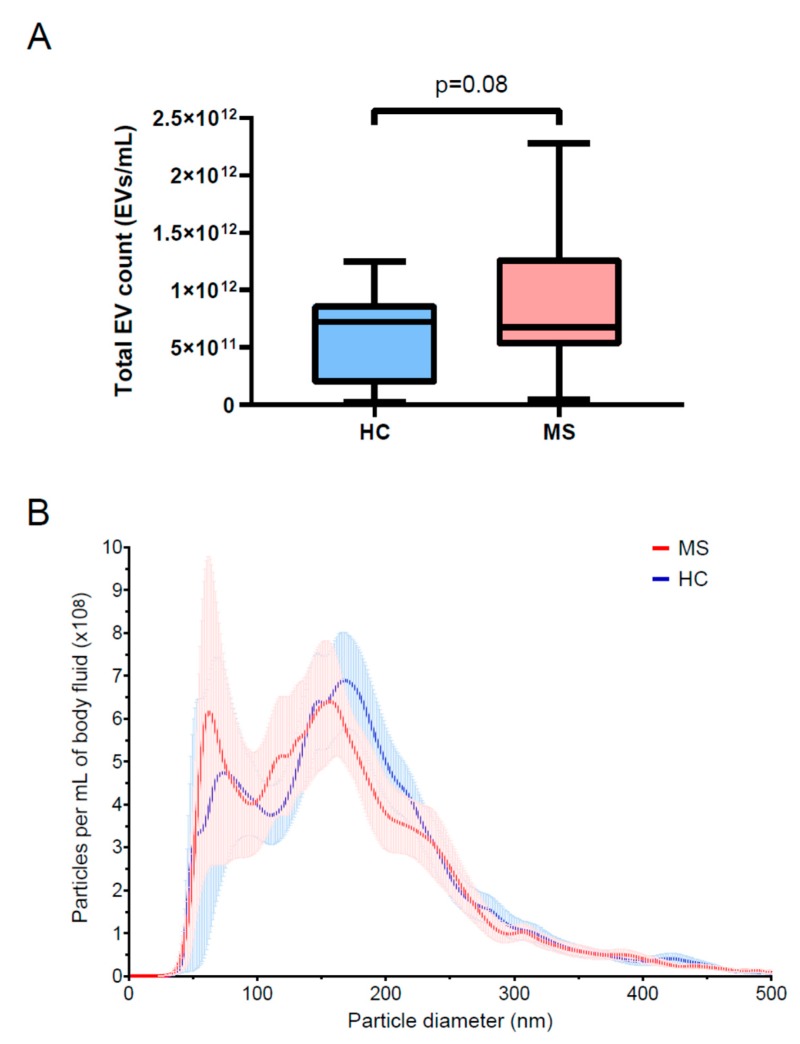
MS and controls do not differ in circulating EV number and size distribution. We compared the concentrations of EVs in serum from MS patients and controls and found no significant difference between the two groups (**A**). We also noted no significant difference in the size distribution of EVs between the two groups (**B**).

There was no significant difference in EV concentration by NTA between the two groups (Figure 2A). The size characteristics of the isolated EVs as analyzed by NTA also appeared similar between the two groups (Figure 2B).

## 4. Discussion

This study utilized a prospectively collected cohort of RRMS patients and controls to discover and validate an alteration in the concentrations of innate immunity-related molecules—TLR3 and TLR4-—in circulating EVs of MS patients. This novel finding indicates the potential role of circulating EVs as a platform for biomarker discovery in MS and also suggests the need to further explore the role of TLRs and innate immunity in MS.

TLRs are pattern recognition receptors that are expressed either on the cell surface or intra-cellularly and sense characteristic molecules associated with pathogens such as bacteria, viruses, or fungi [21]. While the role of TLRs in MS and experimental autoimmune encephalomyelitis (EAE) has received significant attention, there are conflicting data between animal and human studies for several TLRs [17]. TLRs are expressed on both immune cells and several non-immune cells (including all cell types in the central nervous system). TLR3 is localized intra-cellularly and can sense nucleic acids and signal through the TIR-domain-containing adapter-inducing interferon-beta (TRIF)-dependent pathway to increase expression of type I interferons [21]. In mouse models, TLR3 activation appeared to exert protective effects in EAE through enhanced endogenous interferon-beta production and reduced production of Th17 cells [17,22,23]. In a human study utilizing peripheral-blood mononuclear cells from progressive and benign MS patients, stimulation of TLR3 led to increased inflammatory cytokine production in progressive MS patients but upregulation of anti-inflammatory genes in benign MS patients, suggesting a potential protective role in a subset of MS patients [24].

TLR4 is expressed on the cell surface, interacts with lipopeptides and can signal through the Myeloid differentiation primary response protein 88 (MyD88) and TRIF pathways [21]. There has been contradictory data regarding whether TLR4 activation is protective or harmful in EAE. In human studies, increased TLR4 and high-mobility group protein B1 (HMGB1) (a TLR4 agonist) levels were found in cerebrospinal fluid (CSF) and stimulation of microglia and macrophages with lipopolysaccharide (LPS) led to increased production of inflammatory cytokines [17,25].

While all of the aforementioned studies relate to TLR function within cells, the role of TLRs in EVs has not been studied, and it is unclear whether altered TLR levels in the EV compartment have any physiological relevance beyond potentially identifying an imbalance in the cellular compartment. Thus, the altered balance of TLR3 and TLR4 in EVs may represent an altered balance of innate immune signaling processes that could be relevant to the pathogenesis of the disease.

The limitations of our study include a cross sectional design, which precluded us from studying the relationship between levels of TLR3/4 and disease activity; the fact that patients were on variable MS disease-modifying treatments; and a relatively small size. Moreover, this study used total serum EVs that have variable cellular origins; therefore, effects cannot be attributed to any particular cell type but rather reflect organismal-level abnormalities in innate immunity. The main strength of this study was its design that included an unbiased assay that was used to identify candidate biomarkers in a smaller cohort, followed by the use of a targeted assay in a larger cohort.

Future studies with larger sample size, prospective longitudinal design and a more comprehensive panel of TLR molecules could help further define and validate the potential role of EV TLR expression as biomarkers for diagnosis and prognosis in MS. In addition, mechanistic studies involving EVs from MS patients are required to uncover the mechanistic significance of these biomarkers.

## Figures and Tables

**Table 1:** Demographic and disease characteristics of participants.

Characteristic		RRMS	HC
**Number**		18	16
**Age, years (mean ± SD)**		43.9 ± 10.8	39.4 ± 8.9
**Female sex, n (%)**		13 (72.2)	11 (68.5)
**Caucasian Race, n (%)**		16 (88.9)	14 (87.5)
**Expanded Disability Status Scale (EDSS), median (IQR)**		2.0 (1.5)	-
**Disease duration, years (mean ± SD)**		9.8 ± 6.2	-
**Current disease-modifying therapy**	**None**	9	-
	**Glatiramer acetate**	4	-
	**Interferon beta**	3	-
	**Natalizumab**	2	-

**Table 2:** Summary of comparisons of extracellular vesicle (EV) array markers between Multiple Sclerosis (MS) patients and controls.

	MS Rel. Intensity (log2 Mean ± SEM) n = 3	Control Rel. Intensity (log2 Mean ± SEM) n = 3	Total Serum EVs (*p* Values)	Total Serum EVs Normalized by EV Concentration by (Nanoparticle Tracking Analysis) NTA (*p* Values)
CD171	0.35 ± 0.07	1.97 ± 0.24	0.003	0.004
TLR3	3.49 ± 0.87	7.55 ± 0.35	0.011	0.011
Annexin V	0.93 ± 0.63	5.52 ± 0.83	0.012	0.012
CD83	1.46 ± 0.49	4.83 ± 0.64	0.014	0.016
CD3	3.72 ± 0.51	6.75 ± 0.54	0.015	0.018
CD45RA	0.20 ± 0.20	2.94 ± 0.64	0.015	0.018
LAMP-1	0.07 ± 0.07	3.03 ± 0.74	0.016	0.018
a-SYN	0.72 ± 0.43	3.52 ± 0.57	0.017	0.021
CD45RO	1.11 ± 0.51	4.31 ± 0.76	0.025	0.028
CD25	3.12 ± 0.88	6.60 ± 0.59	0.031	0.032
CHRM4	1.45 ± 0.39	3.40 ± 0.49	0.036	0.039
CD197	0.67 ± 0.47	3.85 ± 0.91	0.037	0.04
ADBB2	2.82 ± 0.23	4.38 ± 0.46	0.037	0.04
TSG101	0.07 ± 0.07	2.26 ± 0.73	0.041	0.044
TGFb1	1.93 ± 0.43	4.80 ± 0.85	0.04	0.046
CD206	1.68 ± 1.07	5.66 ± 0.89	0.046	0.046
Alix	1.26 ± 0.64	4.74 ± 0.96	0.039	0.041
HLA ABC	0.16 ± 0.16	3.05 ± 1.38	0.106	0.11
CD8a	0.00 ± 0.00	1.66 ± 0.83	0.118	0.117
CD56	0.00 ± 0.00	1.38 ± 0.69	0.118	0.118
Fas L	0.00 ± 0.00	1.89 ± 0.96	0.118	0.123
TNF RII	0.00 ± 0.00	2.15 ± 1.20	0.147	0.152
TRAIL	0.00 ± 0.00	1.90 ± 1.07	0.151	0.155
CD19	0.00 ± 0.00	1.88 ± 1.10	0.162	0.167
CD45	0.06 ± 0.06	2.25 ± 1.29	0.164	0.17
CD14	0.00 ± 0.00	2.13 ± 1.27	0.169	0.174
LAMP2	0.00 ± 0.00	1.94 ± 1.21	0.183	0.188
CD106	0.00 ± 0.00	1.77 ± 1.12	0.187	0.193
TNF RI	0.30 ± 0.30	2.12 ± 1.15	0.191	0.199
Hsp90	0.00 ± 0.00	1.86 ± 1.23	0.203	0.208
CD4	0.14 ± 0.14	1.62 ± 1.02	0.226	0.233
ICAM-1	0.00 ± 0.00	1.53 ± 1.21	0.277	0.281
CD28	0.67 ± 0.67	2.44 ± 1.36	0.309	0.324
Apo E	0.56 ± 0.56	2.08 ± 1.27	0.337	0.351
Flotillin-1	0.96 ± 0.53	1.91 ± 0.96	0.431	0.435
APP	0.00 ± 0.00	0.00 ± 0.00	nd	nd
Hsp70	0.00 ± 0.00	0.00 ± 0.00	nd	nd
General EV surface markers				
CD81	2.27 ± 0.78	3.95 ± 0.65	0.171	0.167
CD9	2.11 ± 0.17	3.32 ± 0.71	0.172	0.187
CD63	0.32 ± 0.32	1.79 ± 1.25	0.318	0.322

Summary of p-values from unpaired *t*-tests. Green indicate significant differences between controls and MS patients after Benjamini-Hochberg procedure for multiple comparisons using a false discovery rate at 20%.
